# Working Time Arrangements as Potential Risk Factors for Ischemic Heart Disease Among Workers in Denmark: A Study Protocol

**DOI:** 10.2196/resprot.5563

**Published:** 2016-06-22

**Authors:** Harald Hannerz, Ann Dyreborg Larsen, Anne Helene Garde

**Affiliations:** ^1^ National Research Centre for the Working Environment Copenhagen Denmark

**Keywords:** occupational health, prescription drugs, hospital treatment, night worker, long working hours

## Abstract

**Background:**

It has long been suspected that a worker’s risk of developing an ischemic heart disease (IHD) may be influenced by his or her working time arrangements. A multitude of studies have been performed, and special attention has been given to long working hours and nighttime work. The statistical powers of the individual studies have, however, generally been too low to either dismiss or confirm an actual relationship, and meta-analyses of underpowered studies are generally associated with publication bias. Hence, uncertainty remains and whether these factors indeed are related to IHD has yet to be settled.

**Objective:**

This project will test whether the incidences of IHD and usage of antihypertensive drugs among employees in Denmark are independent of weekly working hours and nighttime work. The objective of this paper is to present the intended analyses.

**Methods:**

We will link individual participant data from the Danish labor force survey, 1999–2013, to data on socioeconomic status, industry, emigrations, redeemed prescriptions, hospitalizations, and deaths from registers covering the entire population of Denmark. The study will include approximately 160,000 participants, who will be followed through the registers, from the time of the interview until the end of 2014, for first occurrence of IHD and for antihypertensive drug treatment. We will use Poisson regression to analyze incidence rates as a function of nighttime work and of weekly working hours.

**Results:**

We expect results to be ready in mid-2017.

**Conclusions:**

To our knowledge, this will be the largest study ever of its kind. It will, moreover, be free from hindsight bias, since the hypotheses, inclusion criteria, significance levels, and statistical models will be completely defined and published before we are allowed to link the exposure data to the outcome data.

## Introduction

### Background

Our project will look at rates of ischemic heart disease (IHD) among Danish employees as a function of weekly working hours and nighttime work.

From the viewpoint of cardiovascular risk factors, there appear to be both advantages and disadvantages of long working hours and nighttime work.

One of the advantages of nighttime work is that it usually eliminates exposure to rush hour commuting stress, both to and from work; such stress has been associated with psychological strain [1,2], increased blood pressure [3], and increased rates of acute myocardial infarction [4,5]. Moreover, a survey of more than 400 people in United Kingdom indicated that 44% of people believed that rush hour traffic was the single most stressful part of their life [6]. Daytime workers may reduce their exposure to rush hour traffic, at least in one direction, by choosing to work long hours.

Another potential advantage of nighttime work and long working hours is that they can generate extra income, compared with ordinary daytime work, and thereby reduce the risk or intensity of financial strain. An increased income has been associated with a decreased risk of IHD [7], while financial strain has been associated with an increased risk of hypertension [8] and acute myocardial infarction [9].

The disadvantage of long working hours and nighttime or shift work is that they usually are associated with short sleep duration, mismatch of circadian rhythm, social disruption, and behavioral changes [10,11], which in turn are associated with an increased risk of IHD [12]. The mechanism may be related to disturbed regulation of inflammatory, metabolic, and cardiovascular processes. Thus, short-term sleep restriction increases heart rate [13] and blood pressure [14], changes immune function [13], increases C-reactive protein concentration [13], and decreases glucose tolerance and sympathetic nervous system activity [15].

The evidence of an association between night or shift work and IHD has been reviewed by Frost et al [16], Ha et al [17], and Vyas et al [18]. Frost et al [16] reviewed 14 research papers and concluded that “the available evidence concerning the influence of the type and duration of shift work, as well as sex, on the risk of IHD is too limited to permit any conclusions on these issues." In a meta-analysis that combined the results from 8 studies, Ha et al estimated the effect of night or shift work versus ordinary daytime work, first at a rate ratio (RR) equal to 1.17 (95% CI 1.00–1.37) and then, after adjustment for publication bias, at RR=1.12 (95% CI 0.94–1.33) [17].

Vyas et al [18] reviewed 35 research papers and used the results of the papers in meta-analyses, which estimated the RR of night or shift work versus ordinary daytime work at 1.24 (95% CI 1.10–1.39) for coronary events, 1.23 (95% CI 1.15–1.31) for acute myocardial infarction, and 1.05 (95% CI 1.01–1.09) for ischemic stroke. However, 87% (1,750,565/2,011,935) of the participants in the meta-analyses came from ecologic studies, which of course makes the estimates less certain than their confidence intervals imply.

Virtanen et al [19] investigated the relationship between long working hours and coronary heart disease in a systematic review and meta-analysis, which included results from 12 research papers. The RR for coronary heart disease among workers with “long" versus “normal" working hours was estimated at 1.80 (95% CI 1.42–2.29) when all studies were included and 1.39 (95% CI 1.12–1.72) when restricted to prospective studies. The cut points for long weekly working hours ranged from >40 to >65 hours.

The reviews by Vyas et al [18] and by Virtanen et al [19] suggested that the disadvantages of nighttime work and long working hours tend to outweigh the advantages of such work time arrangements. However, since the meta-analyses only included published studies, we cannot rule out publication bias. The risk of publication bias is especially high if the included studies are too small to be of interest unless the results are statistically significant [20], and this is definitely the case with the studies that Virtanen et al [19] included in their meta-analysis.

Kivimaki et al [21] conducted a more reliable meta-analysis of the relationship between long working hours and IHD, in which they mitigated the risk of publication bias by conducting and inserting a series of new and hitherto unpublished studies. By combining the results from the unpublished studies with corresponding results from published studies, they obtained the following RRs for long versus normal (35–40) working hours a week: 1.02 (95% CI 0.91–1.15) for 41–48 hours, 1.07 (95% CI 0.92–1.24) for 49–54 hours, and 1.08 (95% CI 0.92–1.24) for >54 working hours. These RRs are most probably the best estimates ever of the strength of association between long working hours and coronary heart disease. We should, however, keep in mind that meta-analyses that include published results never can be done blinded. The researchers often know the literature before they formulate inclusion criteria and, however systematic and objective they attempt to be, it can never be ruled out that the selection of papers as well as the selection of estimates within the papers might be influenced by their a priori expectations. From this viewpoint, it would be good to know whether the null finding by Kivimaki et al would be reproduced if it were tested in an equally sized blinded study, that is, a study in which the inclusion criteria, hypotheses, and statistical models were completely specified and published before any results were available.

Kivimaki et al [21] based their RRs on a total of 2481 new cases among 129,301 participants. Our project has access to person-based data on work time arrangements for approximately 160,000 employees with long or normal working hours, which, upon follow-up, we expect to yield approximately 3200 new cases of IHD. The exposure data of the project have not been linked to health data before, and this provides an opportunity to study the relationship between work time arrangements and subsequent IHD, with a remarkably high statistical power, completely blinded.

### Aims and Hypotheses

We want to know whether the incidence of antihypertensive drug usage and the incidence of hospital treatment or death due to IHD are independent of weekly working hours and nighttime work among full-time employees in Denmark, and will address these research questions in a series of nested hypothesis tests (Textbox 1).

**Hypotheses to be tested.**

1. The incidence of antihypertensive drug usage and the incidence of hospital treatment or death due to ischemic heart disease (IHD) among full-time employees in Denmark are prospectively independent of weekly working hours, as well as interaction between weekly working hours and each of the following variables: socioeconomic status, sex, and nighttime work.

1.1. The incidence of hospital treatment or death due to IHD is prospectively independent of weekly working hours, as well as interaction between weekly working hours and each of the following variables: socioeconomic status, sex, and nighttime work.

1.1.1. The prospective association between weekly working hours and incidence of hospital treatment or death due to IHD is independent of socioeconomic status.

1.1.2. The prospective association between weekly working hours and incidence of hospital treatment or death due to IHD is independent of sex.

1.1.3. The prospective association between weekly working hours and incidence of hospital treatment or death due to IHD is independent of nighttime work.

1.1.4. The incidence of hospital treatment or death due to IHD is prospectively independent of weekly working hours when we disregard interaction effects.

1.2. The incidence of antihypertensive drug usage is prospectively independent of weekly working hours, as well as interaction between weekly working hours and each of the following variables: socioeconomic status, sex, and nighttime work.

1.2.1. The prospective association between weekly working hours and incidence of antihypertensive drug usage is independent of socioeconomic status.

1.2.2. The prospective association between weekly working hours and incidence of antihypertensive drug usage is independent of sex.

1.2.3. The prospective association between weekly working hours and incidence of antihypertensive drug usage is independent of nighttime work.

1.2.4. The incidence of antihypertensive drug usage is prospectively independent of weekly working hours when we disregard interaction effects.

2. The incidence of antihypertensive drug usage and the incidence of hospital treatment or death due to IHD among full-time employees in Denmark is prospectively independent of nighttime work, as well as interaction between nighttime work and each of the variables socioeconomic status and sex.

2.1. The incidence of hospital treatment or death due to IHD is prospectively independent of nighttime work, as well as interaction between nighttime work and each of the variables socioeconomic status and sex.

2.1.1. The prospective association between nighttime work and incidence of hospital treatment or death due to IHD is independent of socioeconomic status.

2.1.2. The prospective association between nighttime work and incidence of hospital treatment or death due to IHD is independent of sex.

2.1.3. The incidence of hospital treatment or death due to IHD is prospectively independent of nighttime work when we disregard interaction effects.

2.2. The incidence of antihypertensive drug usage is prospectively independent of nighttime work, as well as interaction between nighttime work and each of the variables socioeconomic status and sex.

2.2.1. The prospective association between nighttime work and incidence of antihypertensive drug usage is independent of socioeconomic status.

2.2.2. The prospective association between nighttime work and incidence of antihypertensive drug usage is independent of sex.

2.2.3. The incidence of antihypertensive drug usage is prospectively independent of nighttime work when we disregard interaction effects.

We will set the overall significance level for the effect of weekly working hours at .05 and we will set the overall significance level for the effect of nighttime work at .05. We will solve the multiple testing problems by the following strategy:

- A null hypothesis at the first level will be rejected if either of its two second-level null hypotheses is rejected.
- A null hypothesis at the second level will be rejected if the *P* value of its statistical test is ≤.025.
- A null hypothesis at the third level will be rejected if (1) its associated second-level null hypothesis is rejected and (2) the *P* value of its statistical test is ≤.025.

Hospital treatment or death due to IHD is the primary outcome of the study, and a statically significant association with this outcome would afford direct statistical evidence of an association with IHD.

Hypertension plays an important role in the etiology of IHD [22], and relative rates of antihypertensive drug usage have been shown to be highly correlated with relative rates of IHD among occupational groups in Denmark [23,24]. We will therefore regard results obtained for antihypertensive drug usage as indirect statistical evidence of an association with IHD if they are statistically significant and show a similar pattern to the results obtained for hospital treatment or death due to IHD.

## Methods

### Ethics Approval

The study will comply with The Act on Processing of Personal Data, Denmark (Act No. 429 of May 31, 2000), which implements the European Union (EU) Directive 95/46/EC on the protection of individuals. The data usage is approved by the Danish Data Protection Agency, file number 2001-54-0180. The ethical aspect of the project was examined and approved by Statistics Denmark, account number 704291.

### Data Material

The data base of the project will consist of interview data from the Danish Labour Force Survey 1999–2013, which are linked to data from the central person register [25], the employment classification module [26], the national patient register [27], the cause of death register [28], and the national prescription register [29]. The linkage will be based on the participants’ personal identification numbers.

The Danish Labour Force Survey has been conducted since 1994, in accordance with EU directives, which apply to all member states of the EU. It is based on random samples of 15- to 74-year-old people in the Danish population. The samples are drawn quarterly and the participants are invited to be interviewed 4 times over a period of one and a half years. The structured interviews, which are done by telephone, cover various aspects of labor market participation, including specifications on working hours and work schedules [30]. The response rate is currently 65% [30].

The central person register contains information on sex, addresses, and dates of birth, death, and migrations for every person who is or has been an inhabitant of Denmark sometime between 1968 and the present. A person’s socioeconomic status (SES), occupation, and industry have been registered annually in the employment classification module since 1975. The national hospital register has existed since 1977 and contains data from all public hospitals in Denmark (>99% of all admissions). From 1977 to 1994, the register only included inpatients, but from 1995 it has also covered outpatients and emergency ward visits. Since 1994, the diagnoses have been coded according to *International Classification of Diseases, Tenth Revision* (ICD-10) [31]. The national prescription register covers all redeemed prescriptions at pharmacies in Denmark since 1995, and the products are coded in accordance with the Anatomical Therapeutic Chemical Classification System (ATC).

### Exposure Variables

The labor force surveys gather person-based information on weekly working hours, calculated by adding the hours worked in secondary jobs to the ones worked in a primary job. The participants are asked first how many hours they usually work and then how many hours they worked during the reference week (a predetermined work week, which occurred 1–4 weeks prior to the interview). They are also asked whether and to what extent they work at night. The questions used to gather this information have changed slightly with time. Before 2001, there was no mention of whether meal breaks should be counted as working hours. During 2001–2006, all participants were instructed to exclude meal breaks when they counted their work hours. As of 2007, the time used for meal breaks is to be counted if the person was paid while eating and is to be excluded otherwise. Another peculiarity that was introduced in 2007 is that the participants are asked whether the weekly working hours vary a lot or there are other reasons that make it difficult to provide a meaningful estimate of usual weekly working hours. If they answer “yes" to any of these questions, then “average working hours" is to be used as a proxy for “usual working hours."

Before 2001, the participants were simply asked whether they worked at night, but from 2001 onward the question has been whether they worked at night during the last 4 weeks. Until 2006 the response categories were “yes, regularly," “yes, occasionally," and “no, never". From 2007 onward the response categories were expanded to “yes, regularly" (ie, more than half of the working days in the last 4 weeks), “yes occasionally" (ie, at least once within the last 4 weeks, but less than half of the working days), and “no, not within the last 4 weeks."

We will disregard the changes in the data collection routines in the primary analyses of this project. We will define the exposure variables as follows.

#### Weekly Working Hours

In keeping with Kleppa et al [32] and Hannerz and Albertsen [33], we will treat working hours as a categorical variable, with 32–40 hours representing normal weekly working hours, 41–48 hours representing overtime work that lies within the limits of the European working time directive, and 49–100 hours representing overtime work beyond the threshold of the directive. We will base the categorization on the person’s usual working hours.

#### Nighttime Work

Participants who responded with either “yes, regularly" or “yes, occasionally" to the question about nighttime work will be defined as being exposed and those who responded with “no..." will be defined as being unexposed to nighttime work.

### Clinical End Points

The primary end point is hospital treatment or death, with IHD as the principal diagnosis or cause of death, respectively. The case definition includes the following ICD-10 codes: I20 angina pectoris, I21 acute myocardial infarction, I22 subsequent myocardial infarction, I23 certain current complications following acute myocardial infarction, I24 other acute IHDs, I25 chronic IHD. The secondary end point is redemption of a prescription for antihypertensive drugs. The following ATC codes are included: C02 antihypertensives, C03 diuretics, C07 alpha- and beta-blockers, C08 calcium channel blockers, and C09 angiotensin-converting enzyme inhibitors and angiotensin-II antagonists.

### Follow-Up and Inclusion Criteria

The participants will be followed from the beginning of the calendar year that succeeds that of their baseline interview. The follow-up will end at the time the participant is diagnosed with IHD, emigrates, or dies, or the end of the study period (December 31, 2014), whichever comes first. To be eligible for inclusion, they should be between 21 and 59 years old at the start of the follow-up period and employed with ≥32 weekly working hours at the time of the interview. People who received hospital treatment for IHD during the calendar year of the interview will be excluded from the IHD analysis. People who redeemed a prescription for antihypertensive drugs during the calendar year of the interview will be excluded from the antihypertensive drug analysis.

### Primary Analysis

We will use Poisson regression to analyze incidence rates of hospital treatment or death due to IHD as a function of weekly working hours (32–40, 41–48, or >48 hours/week), nighttime work (yes vs no), sex, age (10-year classes), calendar time (2000–2004, 2005–2009, or 2010–2014), time passed since start of follow-up (0–4, 5–9, or ≥10 years), employment in the health care industry (yes vs no), and SES (low, medium, high, or unknown). Age, calendar time, and time passed since start of follow-up will be treated as dynamic (time-varying) variables. The remaining variables will be fixed at baseline (the calendar year of the interview). The logarithm of person-years at risk will be used as offset. People who participated in more than one interview will be classified in accordance with the responses given in their first interview. Later interviews will be disregarded.

We will retrieve information on occupation and industry from the employment classification module, and refer it to the status during the calendar year of the baseline interview. Industries were coded in accordance with the Statistics Denmark classification DB93 [34] in 1999–2002, DB03 [35] in 2002–2007, and DB07 [36] in the calendar years 2008–2013. Occupations were coded in accordance with DISCO-88 (the Danish version of the International Standard Classification of Occupations, ISCO-88) [37] in the calendar years 1999–2009 and DISCO-08 (the Danish version of ISCO-08) [38] in the calendar years 2010–2013.

We will code the variable “employment in the health care industry" as “yes" if the 3-digit industrial code of DB93 or DB03 equals 851 or the 2-digit code of DB07 equals 86.

We will base SES on the participant’s occupation and will code it as “high," “medium," or “low" in accordance with the 3-class version of the European Socio-economic Classification (ESeC). The coding will be performed in accordance with the SAS (SAS Institute) programming statements shown in Textbox 2.

**SAS programming statements for occupations coded according to the Danish version of the International Standard Classification of Occupations (DISCO-88) and socioeconomic class (SES) coded according to the European Socio-economic Classification (ESeC).**

/* SES classification (ESeC three class version) of employees by use of DISCO-88 */

if '1' le substr(DISCO_88, 1, 1) le '2' then SES = "High";

if '3' le substr(DISCO_88, 1, 1) le '4' then SES = "Medium";

if '5' le substr(DISCO_88, 1, 1) le '9' then SES = "Low";

if '31' le substr(DISCO_88, 1, 2) le '32' or substr(DISCO_88, 1, 3) in ('334', '342', '344', '345', '348', '521') then SES = "High";

if substr(DISCO_88, 1, 3) = '731' then SES = "Medium";

if substr(DISCO_88, 1, 3) in ('413', '414', '421', '422') then SES = "Low";

/* SES classification (ESeC three class version) of employees by use of DISCO-08 */

if '1' le substr(DISCO_08, 1, 1) le '2' then SES = "High";

if '3' le substr(DISCO_08, 1, 1) le '4' then SES = "Medium";

if '5' le substr(DISCO_08, 1, 1) le '9' then SES = "Low";

if substr(DISCO_08, 1, 3) in ('311', '312', '314', '315', '321', '322' '323') then SES = "High";

if substr(DISCO_08, 1, 3) in ('224', '742') then SES = "Medium";

if substr(DISCO_08, 1, 2) = '42' or substr(DISCO_08, 1, 3) = '432' then SES = "Low";

#### Statistical Models and Tests to Analyze Effects of Weekly Working Hours

The full model will include the following covariates: calendar time, time passed since start of follow-up, employment in the health care industry, age, sex, SES, nighttime work, working hours, working hours × sex, working hours × SES, and working hours × nighttime work. We will use the parameter estimates obtained with the full model to calculate RRs for incident use of antihypertensive drugs and for hospitalization or death due to IHD as a function of weekly working hours, by sex, SES, and nighttime work. We will consider the following contrasts: 41–48 versus 32–40 working hours/week, and >48 versus 32–40 working hours/week. The results will be presented as outlined in Table 1.

**Table 1:** Dummy table for reporting the RR^a^ with 95% CI for incident use of antihypertensive drugs and hospitalization or death due to IHD^b^ as a function of weekly working hours among Danish employees during 2000–2014, stratified by sex, socioeconomic status, and night shift status.

Worker subgroups		Weekly working hours	Antihypertensive drugs			Hospitalization or death due to IHD		
			Cases	RR	95% CI	Cases	RR	95% CI
**Sex**							
	Male	>48						
		41–48						
		32–40		1.00	–		1.00	–
	Female	>48						
		41–48						
		32–40		1.00	–		1.00	–
**Socioeconomic status**									
	Low	>48						
		41–48						
		32–40		1.00	–		1.00	–
	Medium	>48						
		41–48						
		32–40		1.00	–		1.00	–
	High	>48						
		41–48						
		32–40		1.00	–		1.00	–
	Unknown	>48						
		41–48						
		32–40		1.00	–		1.00	–
**Nighttime work**							
	Yes	>48						
		41–48						
		32–40		1.00	–		1.00	–
	No	>48						
		41–48						
		32–40		1.00	–		1.00	–

^a^RR: rate ratio.

^b^IHD: ischemic heart disease.

We will test hypotheses 1.1 and 1.2 by use of likelihood ratios comparing the full model with a model containing only the following covariates: calendar time, time passed since start of follow-up, employment in the health care industry, age, sex, SES, and nighttime work.

We will test hypotheses 1.1.1 and 1.2.1 by use of likelihood ratios comparing the full model with a model containing only the following covariates: calendar time, time passed since start of follow-up, employment in the health care industry, age, sex, SES, nighttime work, working hours, working hours × sex, and working hours × nighttime work.

We will test hypotheses 1.1.2 and 1.2.2 by use of likelihood ratios comparing the full model with a model containing only the following covariates: calendar time, time passed since start of follow-up, employment in the health care industry, age, sex, SES, nighttime work, working hours, working hours × SES, and working hours × nighttime work.

We will test hypotheses 1.1.3 and 1.2.3 by use of likelihood ratios comparing the full model with a model containing only the following covariates: calendar time, time passed since start of follow-up, employment in the health care industry, age, sex, SES, nighttime work, working hours, working hours × sex, and working hours × SES.

We will test hypotheses 1.1.4 and 1.2.4 by use of likelihood ratios comparing a model containing only the covariates calendar time, time passed since start of follow-up, employment in the health care industry, age, sex, SES, nighttime work, and working hours with a model containing only the covariates calendar time, time passed since start of follow-up, employment in the health care industry, age, sex, SES, and nighttime work.

#### Statistical Models and Tests to Analyze Effects of Nighttime Work

The full model will include the following covariates: calendar time, time passed since start of follow-up, employment in the health care industry, age, sex, SES, working hours, nighttime work, nighttime work × sex, and nighttime work × SES. We will use the parameter estimates obtained with the full model to calculate RRs for incident use of antihypertensive drugs and hospitalization or death due to IHD as a function of nighttime work, by sex and SES. The results will be presented as outlined in Table 2.

**Table 2:** Dummy table for reporting the RR^a^ with 95% CI for incident use of antihypertensive drugs and hospitalization or death due to IHD^b^ as a function of nighttime work among Danish employees during 2000–2014, stratified by sex and socioeconomic status.

Worker subgroups		Nighttime work	Antihypertensive drugs			Hospitalization or death due to IHD		
			Cases	RR	95% CI	Cases	RR	95% CI
**Sex**								
	Male	Yes						
		No		1.00	–		1.00	–
	Female	Yes						
		No		1.00	–		1.00	–
**Socioeconomic status**								
	Low	Yes						
		No		1.00	–		1.00	–
	Medium	Yes						
		No		1.00	–		1.00	–
	High	Yes						
		No		1.00	–		1.00	–
	Unknown	Yes						
		No		1.00	–		1.00	–

^a^RR: rate ratio.

^b^IHD: ischemic heart disease.

We will test hypotheses 2.1 and 2.2 by use of likelihood ratios comparing the full model with a model containing only the following covariates: calendar time, time passed since start of follow-up, employment in the health care industry, age, sex, SES, and working hours.

We will test hypotheses 2.1.1 and 2.2.1 by use of likelihood ratios comparing the full model with a model containing only the following covariates: calendar time, time passed since start of follow-up, employment in the health care industry, age, sex, SES, nighttime work, working hours, and working hours × sex.

We will test hypotheses 2.1.2 and 2.2.2 by use of likelihood ratios comparing the full model with a model containing only the following covariates: calendar time, time passed since start of follow-up, employment in the health care industry, age, sex, SES, nighttime work, working hours, and working hours × SES.

We will test hypotheses 2.1.3 and 2.2.3 by use of likelihood ratios comparing a model containing the covariates calendar time, time passed since start of follow-up, employment in the health care industry, age, sex, SES, nighttime work, and working hours with a model containing only the covariates calendar time, time passed since start of follow-up, employment in the health care industry, age, sex, SES, and working hours.

### Power Calculations

To estimate the statistical power of the hypothesis tests, we first needed to estimate the expected number of cases in the various exposure categories.

To obtain such estimates, we followed the entire population of 21- to 59-year-old employees in Denmark from January 1, 2000 and onward in the same registers that we will use to follow up the samples of this project. While discounting prevalent cases (those who had experienced the clinical end point of the study during the calendar year 1999), we noted all new cases that occurred and tabulated those against the time that had passed since the start of the follow-up. Table 3 gives the results, where the values for the first 13 years of IHD cases and the first 12 years of antihypertensive drug cases are based on actual data, whereas we extrapolated the values for the remaining years by use of a second-degree Taylor polynomial.

**Table 3:** Cumulative percentage of new cases among employees in Denmark aged 21–59 years at baseline, as a function of time passed since start of follow-up (January 1, 2000).

Years of follow-up	Hospitalization or death due to IHD^a^		Antihypertensive drugs	
	Men	Women	Men	Women
1	0.34	0.14	1.41	2.38
2	0.65	0.26	2.89	4.63
3	0.98	0.41	4.44	6.76
4	1.32	0.55	6.15	8.98
5	1.67	0.70	8.07	11.23
6	2.03	0.86	10.01	13.38
7	2.39	1.04	12.14	15.62
8	2.76	1.22	14.37	17.89
9	3.14	1.41	16.55	20.05
10	3.53	1.63	18.63	22.02
11	3.98	1.88	20.70	23.92
12	4.42	2.13	22.69	25.75
13	4.83	2.36	24.64	27.54
14	5.21	2.58	26.56	29.32
15	5.58	2.80	28.48	31.09

^a^IHD: ischemic heart disease.

Since the sample of participants who were interviewed in calendar year *n* will be followed for 2014– *n* years, the expected percentages of new cases in that sample can be found in the row headed by 2014– *n* years of follow-up, and so forth.

By relating the frequency distribution of the participants stratified by calendar year of interview, sex, and exposure category to the percentages given in Table 3, we finally obtained the expected numbers of cases that were needed to calculate the power (see Table 4).

**Table 4:** Expected number of new cases under the null hypothesis.

Type of exposure	Level	No. of participants	Expected no. of IHD^a^ cases	Expected no. of antihypertensive drug cases
Night shifts	Yes	20,337	439	2924
	No	137,521	2786	21,068
Weekly working hours	>48	9734	210	1304
	41–48	15,872	297	2082
	32–40	132,252	2718	20,606

^a^IHD: ischemic heart disease.

**Figure 1:**
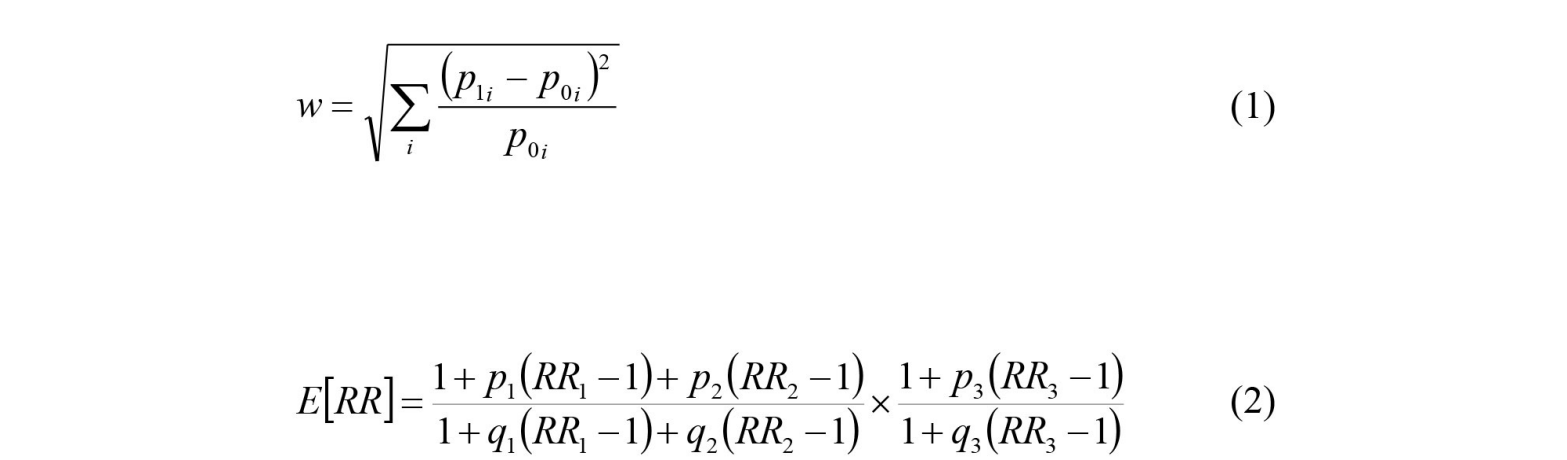
Equation (1): calculation of Cohen effect size w, where *p*_0 *i*_ and *p*_1 *i*_ are the expected proportions of cases that fall into exposure category *i* under the null hypothesis and the alternative hypotheses. Equation (2): calculation of expected rate ratio, E[RR], where *RR*_1_ is the rate ratio for ischemic heart disease (IHD) among employees in the body mass index (BMI) category 25≤BMI<30 versus BMI<25 kg/m^2^, *RR*_2_ is the rate ratio for IHD among employees in the category BMI≥30 versus BMI<25 kg/m^2^, and *RR*_3_ is the rate ratio for IHD among smoking versus nonsmoking employees. The parameters *p*_1_ and *q*_1_ are the proportions of employees who belong to the category 25≤BMI<30 kg/m^2^, *p*_2_ and *q*_2_ are the proportions of employees who belong to the category BMI≥30 kg/m^2^, and *p*_3_ and *q*_3_ are the proportions of smokers among employees with and without nighttime work.

Since our hypotheses will be evaluated by use of chi-square distributed likelihood ratio tests, we have chosen to depict the statistical power as a function of Cohen w, which is an effect size defined by equation (1) (Figure 1). Cohen classified w=0.1 as a small effect, w=0.3 as a medium effect and w=0.5 as a large effect [39].

**Figure 2:**
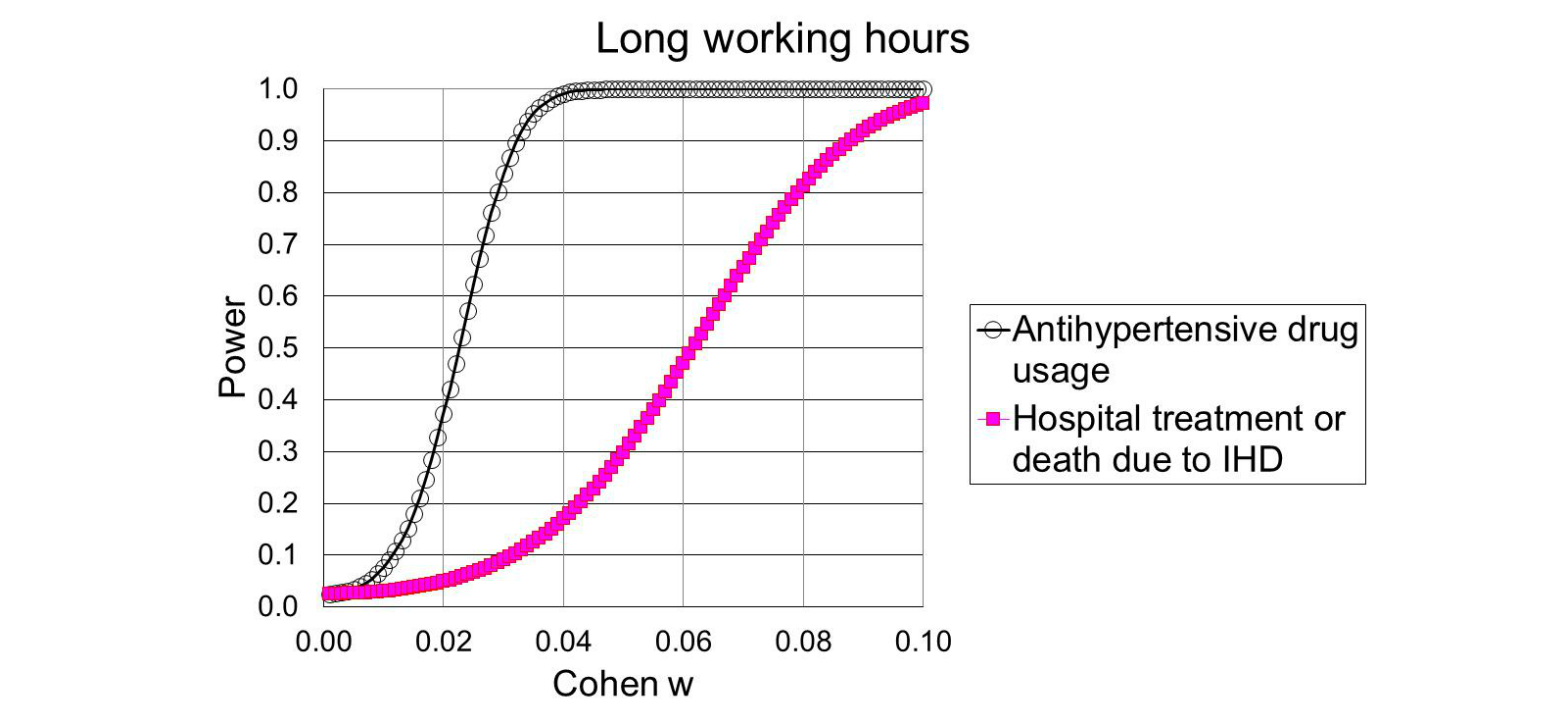
Power to detect that the examined incidences depend on weekly working hours either as a general effect or as an effect of interaction with sex, socioeconomic status, or nighttime work, as a function of Cohen w. IHD: ischemic heart disease.

**Figure 3:**
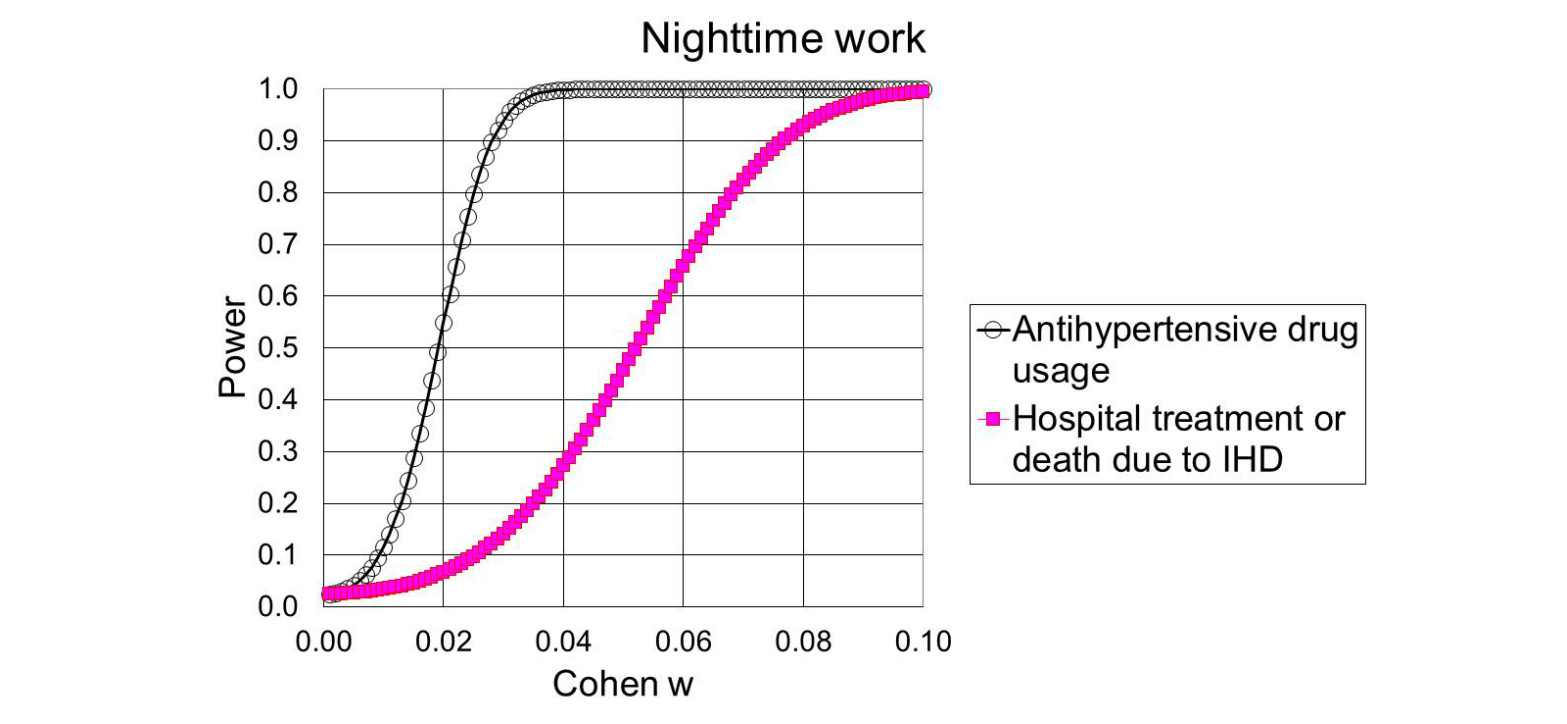
Power to detect that the examined incidences depend on nighttime work either as a general effect or as an effect of interaction with sex or socioeconomic status, as a function of Cohen w. IHD: ischemic heart disease.

Figure 2 shows the statistical power of hypotheses 1.1 and 1.2. Figure 3 shows the power of hypotheses 2.1 and 2.2. The calculations are based on the total number of expected cases and the noncentral chi-square distribution with 12 degrees of freedom for the effects of long working hours (hypotheses 1.1 and 1.2) and 5 degrees for the effects of nighttime work (hypotheses 2.1 and 2.2).

### Sensitivity Analysis 1

Since the questions used to obtain information about nighttime work and weekly working hours were revised in 2001 and then again in 2007, we will perform a sensitivity analysis with the results stratified by calendar period of interview (1999–2000, 2001–2006, and 2007–2013). The end point, covariates, and statistical model of the sensitivity analysis will be the same as the ones used to test hypotheses 1.1.4 and 2.1.3.

### Sensitivity Analysis 2

To ascertain that an observed instance of hospital treatment during the follow-up is a new episode rather than a revisit in a course of treatment that was initiated before baseline, the primary analysis will exclude all workers who were treated for IHD sometime during the calendar year preceding baseline. It will, however, not exclude all former cases of IHD, and it is possible that the estimates of the primary analysis will be affected by nonexcluded workers who were treated for IHD more than 1 year earlier than baseline. We will address this issue with a sensitivity analysis, which will exclude all workers who received hospital treatment for IHD one or more times during a 5-year period prior to baseline. The analysis will include only those who were at least 20 years old and lived in Denmark throughout the 5-year period of interest. In all other respects, the design will be the same as the one used to test hypotheses 1.1.4 and 2.1.3.

### Sensitivity Analysis 3

The actual working hours, that is, the hours worked during the reference week, constitute a well-defined quantity with minimal recall bias. The usual working hours are less well defined, and the way they are understood and remembered might vary between individuals. In spite of this drawback, we chose to base our analysis on the workers’ usual rather than their actual working hours. We did so because some of the participants, by chance, would have worked less than usual during the reference week due to, for example, holidays, vacation, or sickness absence, while others would have worked more than usual due to, for example, a deadline or a temporary staff shortage. Since the usual working hours might be associated with recall bias, we will perform a sensitivity analysis in which we include only participants who belong to the same category according to their actual working hours as they do according to their usual working hours. In all other respects, the design will be the same as the one used to test hypotheses 1.1.4 and 2.1.3. Table 5 gives a -tabulation of the actual and usual working hours.

**Table 5:** Number of economically active 21- to 59-year-old participants, stratified by combinations of actual and usual weekly working hours.

Actual weekly working hours	Usual weekly working hours				
	0–31	32–40	41–48	>48	Total
Missing	187	56	16	19	278
0–31	31,094	32,748	2888	1545	68,275
32–40	2603	82,781	2762	788	88,934
41–48	462	10,981	8250	869	20,562
>48	413	5686	1956	6513	14,568
Total	34,759	132,252	15,872	9734	192,617

### Statistics on Smoking, Overweight, and Obesity

It is recognized that the risk of IHD depends on a person’s body mass index (BMI) and smoking habits. Among Danish employees, the RR for IHD has been estimated at 1.54 for current versus never smokers [40], at 1.41 for 25≤BMI<30 versus BMI<25, and at 2.69 for BMI≥30 versus BMI<25 kg/m^2^[41].

Unfortunately, the Danish Labour Force Survey does not contain any information about the worker’s weight and smoking habits, which makes us unable to control for these factors in our analyses. We therefore wanted to know in what direction and to what extent we can expect the estimates of the project to be influenced by differences in smoking habits and BMI. To shed some light on this issue, we compiled some descriptive statistics on the prevalence of smoking and high BMI in relation to long working hours and nighttime work among a random sample of employees in Denmark (Table 6,Table 7). The sample was drawn in 2010 and the data were collected by use of a mailed questionnaire. The response rate was 48% [42].

**Table 6:** Crude percentages of current smokers, persons with moderate overweight (25≤BMI^a^<30 kg/m^2^), and persons with obesity (BMI≥30 kg/m^2^), by working time arrangement, in a random sample of 20- to 59-year-old employees in Denmark, 2010.

Working time arrangements	Current smoker	25≤BMI<30	BMI≥30
	% (n/N)	% (n/N)	% (n/N)
32–40 working hours/week	22.4 (1205/5383)	33.8 (1821/5383)	12.9 (695/5383)
41–48 working hours/week	20.7 (255/1231)	36.1 (445/1231)	12.8 (157/1231)
>48 working hours/week	21.0 (141/671)	39.6 (266/671)	12.1 (81/671)
Without nighttime work	21.7 (1465/6766)	34.5 (2335/6766)	12.7 (858/6766)
With nighttime work	26.2 (136/519)	38.0 (197/519)	14.5 (75/519)

^a^BMI: body mass index.

**Table 7:** Age (10-year classes) and sex standardized percentages of current smokers, persons with moderate overweight (25≤BMI^a^<30 kg/m^2^), and persons with obesity (BMI≥30 kg/m^2^), by working time arrangement, in a random sample of 20- to 59-year-old employees in Denmark, 2010.

Working time arrangement	Current smoker		25≤BMI<30		BMI≥30		
	%	95% CI	%	95% CI	%	95% CI
32–40 working hours/week	22.6	21.5–23.7	34.7	33.4–35.9	12.9	12.1–13.9
41–48 working hours/week	21.0	18.8–23.5	34.8	32.2–37.5	12.5	10.7–14.5
>48 working hours/week	21.3	18.0–25.2	35.3	31.6–39.5	10.8	8.5–13.7
Without nighttime work	21.6	20.7–22.6	34.5	33.4–35.6	12.7	11.9–13.5
With nighttime work	25.8	22.3–30.0	38.4	34.5–42.8	15.4	12.5–19.0

^a^BMI: body mass index.

Table 7 suggests that the prevalence of smoking, overweight, and obesity tend to be higher among employees who work at night than among those who don’t. To get an idea of the extent to which such differences may influence the RR of IHD between employees with and without nighttime work, we calculated an expected rate ratio, E[RR], under the assumption that the groups are equal in all respects other than the smoking and BMI distributions, using equation (2) (Figure 1).

Our calculations imply that a failure to control for smoking, overweight, and obesity (in a study population in which the prevalences are equal to those given in Table 7) would increase E[RR] of IHD between employees with and without nighttime work by a factor of 1.07.

## Results

We expect results to be ready in mid-2017.

## Discussion

This study protocol provides a detailed description of the hypotheses, inclusion criteria, significance levels, and statistical models of a project designed to estimate prospective associations between different types of work time arrangements and IHD in the general working population of Denmark.

Statistics Denmark randomly sampled the participants in the study from the target population and we have strengthened the prospective design by the strategy to exclude prevalent cases.

For nighttime work and long working hours, the statistical power is sufficient (compare [43]), and the nested hypothesis testing ensures that the probability that statistically significant findings will arise by chance alone is <5%.

Since the design of the project is being peer reviewed and published before the exposure data are linked to health data, we have eliminated hindsight and within-study selection bias. We have also eliminated bias from missing follow-up data, since the clinical end points are ascertained through registers that cover the entire target population.

It has previously been shown that the incidence of IHD is highly dependent on age [44], sex [44], and SES [23,45,46] It has, moreover, been suggested that employment in the health care sector is associated with referral and prescription bias [23,47,48] Our statistical models will mitigate the bias from these factors, by incorporating them as control variables. We can, however, not rule out the possibility of bias due to factors that we cannot control for. For example, the EU Working Time Directive stipulates that night workers are entitled to a free health assessment, prior to their assignment and at regular intervals thereafter, and such a stipulation might be associated with detection bias [49].

Another drawback is that the definition of “night worker" that we use in this project differs from the legal definition that is used in the EU Working Time Directive. According to the Danish implementation of the directive, “nighttime" means any period of at least 7 hours, which includes the period between midnight and 5:00 AM, while “night worker" means “any worker, who, during nighttime, work at least three hours of his daily working time as a normal course" or “any worker who is likely to work at nighttime at least 300 hours during a period of twelve months" [50]. In contrast, our project classifies a participant as a night worker if she or he worked at night either regularly or occasionally during the 4-week period preceding the interview. Prior to 2007, the questionnaire did not specify how to interpret the response categories, but from 2007, “regularly" meant “more than half of the working days in the last 4 weeks," while “occasionally" meant “at least once within the last 4 weeks, but less than half of the working days." Hence, some of the night workers in this project would probably not qualify as night workers in a strictly legal sense.

In conclusion, this is an observational study, which cannot be used to confirm etiological hypotheses. It may, however, confirm that long working hours or nighttime work, or both, are predictors for IHD and thereby lend support to the hypothesis of a causal relationship.

## Abbreviations

ATC: Anatomical Therapeutic Chemical Classification System
BMI: body mass index
DISCO-88: Danish version of the International Standard Classification of Occupations
E[RR]: expected rate ratio
ESeC: European Socio-economic Classification
EU: European Union
ICD-10: International Classification of Diseases, Tenth Revision
IHD: ischemic heart disease
ISCO-88: International Standard Classification of Occupations
RR: rate ratio
SES: socioeconomic status
